# A cuproptosis-related gene expression signature predicting clinical prognosis and immune responses in intrahepatic cholangiocarcinoma detected by single-cell RNA sequence analysis

**DOI:** 10.1186/s12935-024-03251-2

**Published:** 2024-03-02

**Authors:** Hefei Ren, Chang Liu, Cheng Zhang, Hongkun Wu, Jiafeng Zhang, Zhenhua Wang, Lei Chen, Huiquan Wang, Chenghao Shao, Lin Zhou

**Affiliations:** 1 Department of Laboratory Medicine, Shanghai Changzheng Hospital, Naval Medical University, Shanghai, 200003 China; 2 Institute of Aging & Tissue Regeneration, State Key Laboratory of Systems Medicine for Cancer and Stress and Cancer Research Unit of Chinese Academy of Medical Sciences (No. 2019RU043), Ren-Ji Hospital, Shanghai Jiao Tong University School of Medicine (SJTU-SM), Shanghai, 200127 China; 3 Department of Pancreatic-Biliary Surgery, Shanghai Changzheng Hospital, Naval Medical University, Shanghai, 200003 China

**Keywords:** Intrahepatic cholangiocarcinoma, Cuproptosis, Prognostic signature, Single-cell analysis, Immune microenvironment

## Abstract

**Background:**

Cholangiocarcinoma represents a malignant neoplasm originating from the hepatobiliary tree, with a subset of tumors developing inside the liver. Intrahepatic cholangiocarcinomas (ICC) commonly exhibit an asymptomatic presentation, rendering both diagnosis and treatment challenging. Cuproptosis, an emerging regulated cell death pathway induced by copper ions, has garnered attention recently. As cancer cells show altered copper metabolism and comparatively higher copper needs, cuproptosis may play a role in the development of ICC. However, studies investigating this possibility are currently lacking.

**Methods:**

Single-cell and bulk RNA sequence data were analyzed, and correlations were established between the expression of cuproptosis-related molecules and ICC patient survival. Genes with predicting survival were used to create a CUPT score using Cox and LASSO regression and tumor mutation burden (TMB) analysis. The CIBERSORT software was employed to characterize immune cell infiltration within the tumors. Furthermore, immune infiltration prediction, biological function enrichment, and drug sensitivity analyses were conducted to explore the potential implications of the cuproptosis-related signature. The effects of silencing solute carrier family 39 member 4 gene (SLC39A4) expression using siRNA were investigated using assays measuring cell proliferation, colony formation, and cell migration. Key genes of cuproptosis were detected by western blotting.

**Results:**

The developed CUPT score divided patients into high and low CUPT score groups. Those with a low score had significantly better prognosis and longer survival. In contrast, high CUPT scores were associated with worse clinical outcomes and significantly higher TMB. Comparisons of the two groups also indicated differences in the immune infiltrate present in the tumors. Finally, we were able to identify 95 drugs potentially affecting the cuproptosis pathway. Some of these might be effective in the treatment of ICC. The in vitro experiments revealed that suppressing the expression of SLC39A4 in ICC cell lines resulted in reduced cell proliferation, colony formation, and cell migration. It also led to an increase in cell death and the upregulation of key genes associated with cuproptosis, namely ferredoxin 1 (FDX1) and dihydrolipoyl transacetylase (DLAT). These findings strongly suggest that this cuproptosis-associated molecule may play a pivotal role in the development and metastasis of ICC.

**Conclusions:**

Changes in the expression of a cuproptosis-related gene signature can predict the clinical prognosis of ICC with considerable accuracy. This supports the notion that cuproptosis influences the diversity and complexity of the immune microenvironment, mutational landscape, and biological behavior of ICC. Understanding this pathway better may hold promise for the development of innovative strategies in the management of this disease.

**Supplementary Information:**

The online version contains supplementary material available at 10.1186/s12935-024-03251-2.

## Introduction

Cholangiocarcinoma (CCA) is a primary malignant tumor caused by the abnormal differentiation and proliferation of biliary epithelial cells. Lesions originating in the bile ducts within the liver parenchyma are referred to as intrahepatic cholangiocarcinoma (ICC). This subset accounts for 20% to 30% of all CCA cases [1]. Clinically, early stages of ICC are asymptomatic or cause mild, uncharacteristic symptoms. Consequently, these tumors are typically diagnosed at an advanced stage, when they cannot be fully resected surgically. As a result, the 5-year survival of ICC patients remains poor. Given that the incidence of these tumors is increasing, the diagnosis and treatment of ICC represents a problem worldwide [2]. In pathologic terms, most ICCs are adenocarcinomas, with a smaller number of squamous cell carcinomas and mucinous carcinomas being detected. The pathogenesis of ICC is unclear, although some studies suggested that cholestasis and persistent biliary tract inflammation may contribute to the development of these malignancies [3]. Currently, surgical intervention remains the primary treatment modality for ICC. However, in cases where surgery is not feasible, adjuvant chemotherapy, targeted therapy, and local therapy options are employed to extend survival [4]. There is an optimistic outlook that a deeper comprehension of the molecular pathogenesis of ICC could lead to the identification of specific therapeutic targets. Recent clinical trials, including individualized treatment plans for immunotherapy, have been built upon this foundation and provide new directions and hope in the diagnosis and treatment of this devastating disease [5–7].

Copper ions function as cofactors at the active sites of various enzymes participating in a variety of physiological processes, including oxidative stress, lipid metabolism, and energy metabolism [8]. Copper can also induce cell death by inducing reactive oxygen species accumulation, apoptosis, proteasome inhibition, and mitochondrial dysfunction [9]. In recognition of this phenomenon, Tsvetkov et al. [10] recently proposed a novel, copper-driven cell death pathway termed “copper death—cuproptosis.” During mitochondrial respiration, cuproptosis can result in direct interactions between copper ions (Cu^2+^ and Cu^+^) and fatty acids and acylated proteins. This process downregulates the expression of iron-sulfur cluster proteins, leading to proteotoxic stress and eventual cell death. Altering copper homeostasis can potentially affect tumor cells through two distinct mechanisms. First, chelating agents binding copper could reduce the availability of the ions in tumor cells, affecting their ability to proliferate and metastasize. Second, manipulating transporter molecules to increase intracellular copper levels can elevate copper concentration inside the cells, subsequently triggering the production of reactive oxygen species and the formation of other toxic molecular complexes that can induce tumor cell death [11, 12].

Despite accumulating evidence that cuproptosis affects tumors, there is currently a lack of studies investigating the role of copper ions in ICC pathogenesis. The objective of this study was to investigate the impact of cuproptosis-related molecules on the prognosis of ICC by analyzing RNA expression data obtained from both single-cell and bulk RNA tumor samples. Using these samples, we specifically investigated the influence of cuproptosis-related signature on the immune cell infiltration and the mutational landscape in ICC. Additionally, we also explored whether potential novel therapeutic drug targets could be identified by analyzing the pharmacological interactions of proteins involved in cuproptosis.

## Materials and methods

### Dataset preparation

Bulk RNA sequence data of ICC samples were accessed from the Cancer Genome Atlas (TCGA) database (https://xenabrowser.net/) and the Gene Expression Omnibus (GEO) database (https://www.ncbi.nlm.nih.gov/geo). A training set of 32 ICC samples was selected from the TCGA dataset and 83 ICC samples from GEO (dataset GSE89749) were used for validation. According to the literature associated with the uploaded GEO dataset [13], we have diligently completed the clinical information of ICC patients within the database. Consequently, we exclusively incorporated patients with comprehensive clinical data into our analysis. Expression data were Log2 transformed. Single-cell sequence data of ICC samples were downloaded from the GEO database. Information derived from five patients with intrahepatic cholangiocarcinoma was analyzed from the GSE138709 dataset [14].

**Fig. 1:**
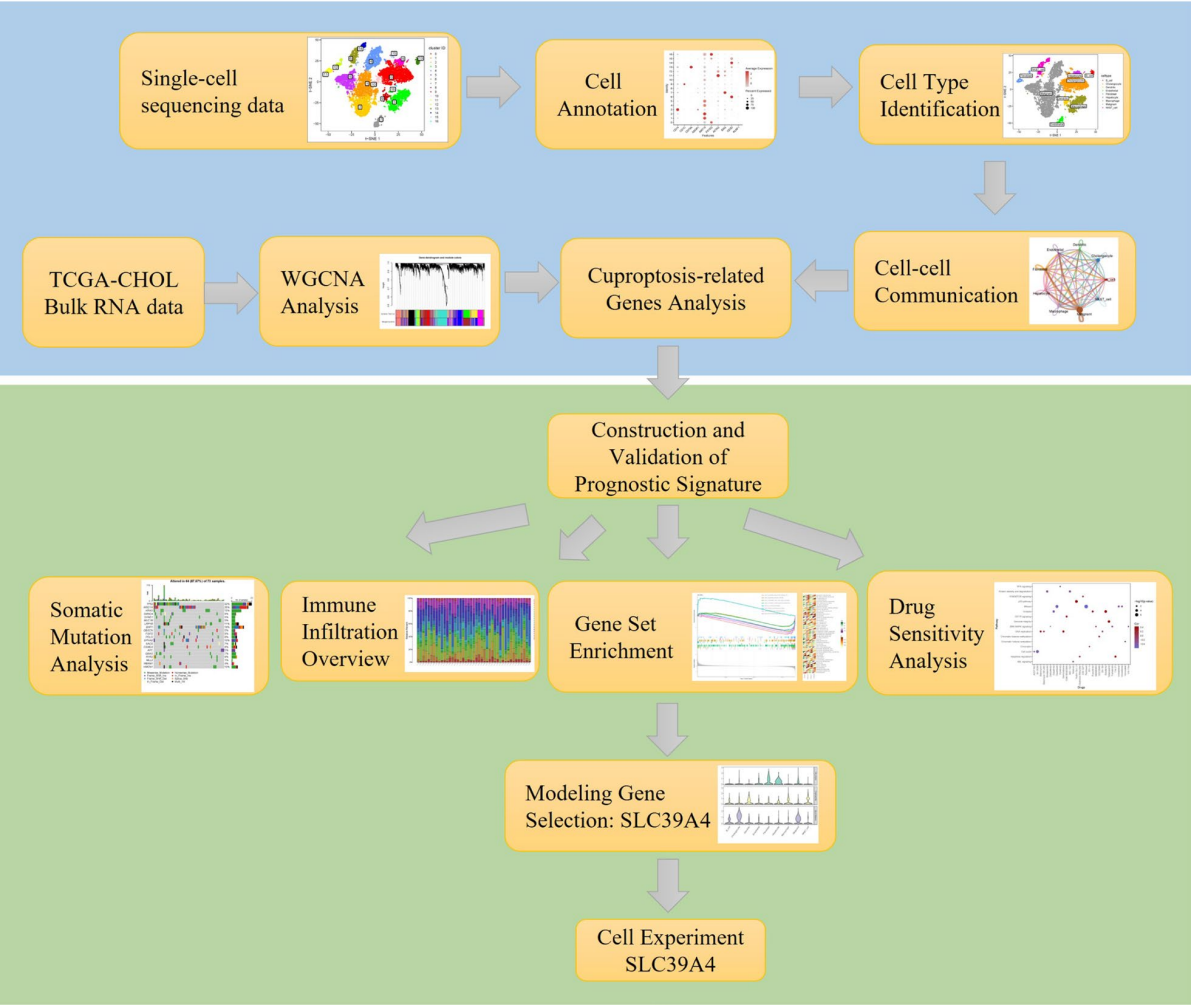
Flow chart of the construction and analysis of the reported prognostic signature

Additional data from the TCGA and GEO portals included details on somatic mutations, clinical information, and survival data. Somatic variations were annotated using the Mutation Annotation Format (MAF) tool within the “maftools” R package [15]. The flowchart of this preparatory work is summarized in Fig. 1.

### Single-cell sequence analysis

The GSE138709 dataset contains individual single cell data derived from five ICC samples. The initial quality control of this raw single-cell data was conducted using the “Seurat” package in R. The data were filtered to contain < 10% mitochondrial genes, < 3% red blood cells, and to include more than 300 genes expressed simultaneously present in at least three cells. To merge these samples, we utilized the “Merge” function of the “Seurat” package. In the next step the “NormalizeData” function of “Seurat” was used to log-normalize gene expression matrices from the included cell data. Finally, the “FindVariableFeatures” function was used to identify highly variable differentially expressed genes.

For dimension reduction, we then used the Harmony algorithm from the “Harmony” R package to eliminate batch effect and generate t-distributed stochastic neighbor embedding (tSNE) [16]. Using the “FindNeighbors” and “FindClusters” functions, we clustered the merged data into 17 cell populations (using the parameters dims = 1:15, resolution = 0.4). We carried out a further tSNE dimensionality reduction by utilizing the “RunTSNE” function.

We then used the signatures identified in the original publication to annotate cell populations [14]. The signature of KRT19 was used to define the malignant cell cluster and the KLRF1 and CD3D signatures were used to define NK cells and T cells, respectively. The ENG signature was used to identify endothelial cells, ASGR1 for hepatocytes and ACTA for fibroblasts. Dendritic cells were detected based on the CD1C signature, while CD79A was used to define the B cell cluster. Finally, cholangiocytes were identified based on the FXYD2 signature. By importing a list of known cuproptosis-related genes and using the “PercentageFeatureSet” function, it was possible to determine the proportion of cuproptosis-related genes present in each cell.

### Analysis of cuproptosis-related genes by single sample gene set enrichment analysis (ssGSEA)

First, we compiled a list of cuproptosis-associated genes by searching the previously published literature [8, 17]. This list, containing 19 known cuproptosis-related genes, was used to calculate a cuproptosis enrichment score. This list consists of 19 well-known cuproptosis-related genes, including NFE2L2, NLRP3, ATP7B, ATP7A, SLC31A1, FDX1, LIAS, LIPT1, LIPT2, DLD, DLAT, PDHA1, PDHB, MTF1, GLS, CDKN2A, DBT, GCSH, and DLST. Subsequently, we calculated a cuproptosis enrichment score using ssGSEA analysis on the TCGA sample dataset [18]. This enrichment score reflects the relative abundance of cuproptosis-related genes within each sample.

### Construction and validation of a cuproptosis-related prognostic signature

Based on cuproptosis-related gene scores, a weighted gene co-expression network analysis (WGCNA) was conducted using the “WGCNA” R package [19]. The strongest predictive features were selected to build a generalized linear model. To minimize overfitting, we employed least absolute and selection operator (LASSO) regression analysis. Cuproptosis-associated genes with prognostic clinical significance were initially identified using univariate logistic regression [20]. In the multivariate logistic regression analysis, we included genes associated with cuproptosis with non-zero LASSO coefficients. Forest plots, providing the graphical representation of the outcomes of the logistic regression analysis, were created using the “ggplot2” R program. The prognostic model was constructed based on the derived information.

To establish the prognostic signature and determine the CUPT score, ICC data from the samples in the TCGA dataset were used as the training cohort, and a risk score, referred to as CUPT score, was calculated using a formula established through multivariate logistic regression analysis. Samples were subdivided into a high and a low CUPT group, using the median expression level of the training CUPT score to discriminate between the two subsets. Survival analysis was performed using the available Kaplan-Meier curves. The validation of the prognostic signature was carried out using ICC samples available in the GEO database.

### Relationship between mutations and Tumor Mutation Burden (TMB) analysis

The genetic mutational landscape of ICC patients was visualized using the somatic mutation data extracted from the TCGA “Masked Somatic Mutation” dataset and from the GSE89749 “Somatic Nonsilent Single Nucleotide Variations and Indels” data [13] using the “maftools” function in R. The top 20 genes exhibiting the highest frequency of mutations were displayed to assess differences in mutations between groups. The TMB value, indicating the number of total somatic mutations per megabase of DNA, is an increasingly recognized quantitative biomarker. Using the median TMB value as threshold, patients were divided into a TMB-high and a TMB-low group. These groups were treated as distinct entities during survival analysis.

### Immune infiltration prediction

To explore the correlation between the appearance of infiltrating immune cell subsets and the prognostic CUPT signature, we used the “cibersort” function in the CIBERSORT program to assess the enrichment of specific immune cells. This analysis enabled us to estimate the composition of the immune infiltrate [21]. We also examined the relationship between various immune cell subsets using CIBERSORT analysis.

### Gene set enrichment analysis

The functional analysis, known as “gene set variation analysis” (GSVA), evaluates the enrichment of gene profiles by utilizing an expression matrix [22]. To investigate the enrichment of specific molecular pathways, we obtained the gene set "h.all.v7.5.1.symbols" from the MSigDB database (http://www.gsea-msigdb.org/gsea/msigdb/index.jsp) and performed the analysis using the "GSVA" function in R. The analysis of the biological function of each individual gene was conducted using gene set enrichment analysis (GSEA) [23], while KEGG pathway analysis was performed using the “clusterProfiler” tool.

### Potential therapeutic strategy in ICC

We combined the gene expression matrix derived from the ICC samples with the drug interaction information available in the Cancer Genome Project (CGP). This database contains information on the actions of 138 anticancer drugs against 727 cell lines. Using the “pRRophetic” R package, we analyzed the correlation between CUPT scores and the half-maximum inhibitory concentrations (IC50s) of drugs effective in inhibiting each cell line [24]. Information from the Genomics of Drug Sensitivity in Cancer database (GDSC, http://www.cancerrxgene.org/downloads) was used to analyze the molecular pathways targeted by these drugs [25].

### Cell culture and siRNA treatment

Two human ICC cell lines, HCCC9810 and RBE, were provided by Shanghai Changzheng Hospital (Shanghai, China). The cells were cultured in DMEM medium containing 10% fetal bovine serum at 37 °C in a 5% CO_2_ containing atmosphere. Silencing RNAs affecting the SLC39A4 gene were synthesized by Genomeditech (Shanghai, China). The cell lives were transfected with this siRNA using the riboFECT CP reagent (RIBOBIO, Shanghai, China), according to the manufacturer’s instructions. The effectiveness of the siRNA treatment was determined using quantitative qRT-PCR performed 48 h after transfections. The 2^−ΔΔ^CT method was utilized to calculate the relative expression of the target genes, using GAPDH as internal standard. All experiments were conducted in triplicate. The primers used to amplify SLC39A4 and GAPDH were purchased from Sangon Biotech (Shanghai, China). Primer sequences are listed in Additional file 2: Table S1.

### Proliferation analysis

To assess the viability of HCCC9810 and RBE cells after siRNA treatment we used the CCK8 reagent (Yeasen, Shanghai, China) according to the manufacturer’s instructions. Cell proliferation was measured at 0, 24, 48, and 72 h after treatment. The OD450 value was determined using a microplate reader (BIO-RAD, USA). The ability of cells to form viable colonies was tested by seeding cells into 6-well tissue culture plates. Developing colonies were detected by fixing the cells using 4% paraformaldehyde for 30 min, followed by Crystal violet staining. Colonies were photographed under a microscope. Cell viability assays and colony forming assays were performed in triplicate.

### Cell death detection

To detect cell death, we collected the cells to be tested and washed them twice with PBS buffer. Subsequently, the cell suspension was transferred to flow cytometry tubes. To each tube, 0.5–1 μl of SYTOX^™^ Green nucleic acid stain (Invitrogen, USA) was added and briefly vortexed. The tubes were then incubated at room temperature, avoiding light, for 30 min. After incubation, 400 μl of PBS buffer was added to each tube, and the cells were analyzed using a flow cytometer.

### Western blot analysis

HCCC9810 and RBE cells were lysed in RIPA buffer (Beyotime Biotechnology, China) containing PMSF (Beyotime Biotechnology) and a phosphatase inhibitor (Epizyme Biotech, China). Protein samples were separated using 12.5% sodium dodecyl sulfate–polyacrylamide gel electrophoresis (SDS-PAGE), and transferred to polyvinylidene fluoride membrane (PVDF) membranes (Invitrogen, USA). Membranes were blocked in 5% skimmed milk for 1 h at room temperature and incubated overnight at 4 °C, using the primary antibodies at dilutions indicated below. After washing the membranes were incubated with the secondary antibody for 1-h. The signal was documented using the ChemiDoc XRS + imaging equipment (BIO-RAD). The primary antibodies and their dilutions used in this study were: anti‐SLC39A4 (1:1000, proteintech, USA), anti‐FDX1 (1:1000, proteintech), anti-DLAT (1:1000, proteintech), and anti‐β-Actin (1:1000, CST, USA).

### Statistical analysis

Statistical analyses were carried out using R × 64–4.2.1. The Wilcoxon rank-sum test was used to examine differences between non-normally distributed variables, while the t-test was used to evaluate differences in quantitative data in normally distributed variables. Differences between more than two groups of variables were explored using one-way analysis of variance and the Kruskal–Wallis test. Spearman analysis was used for the correlation analysis. A *P* < 0.05 was accepted to indicate statistical significance in two-sided test.

## Results

### Comprehensive dissection and clustering of scRNA-seq data from ICC samples

**Fig. 2:**
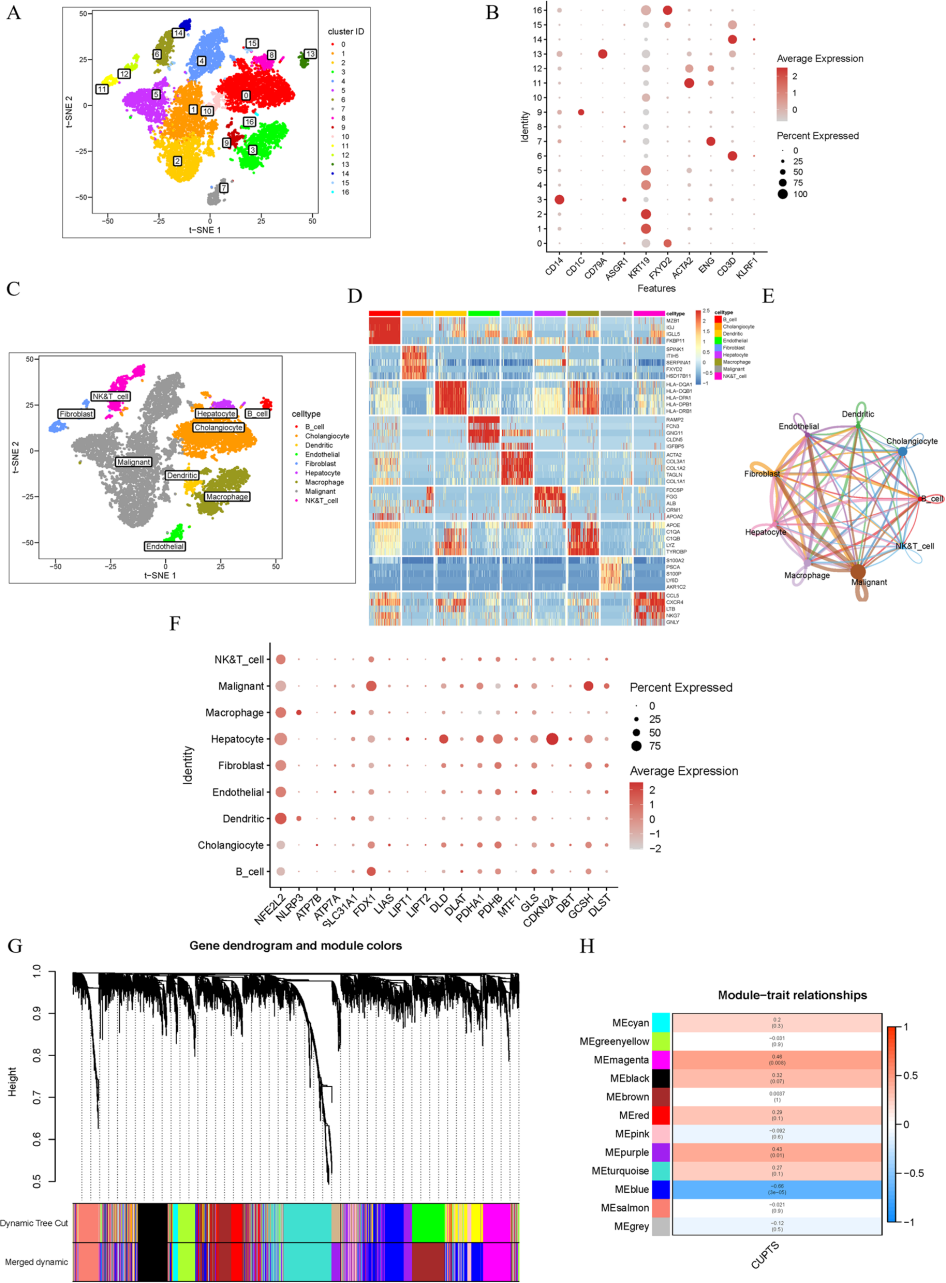
Overview of scRNA data and gene screening. **A** t-SNE plot of cell clusters in ICC. **B** Bubble diagram displaying ICC marker genes in each gene cluster. **C** t-SNE plot of cell types in ICC. **D** Heatmap of differentially expressed genes in different cell types. **E** Analysis of cell–cell communication between various cell types. A cluster is represented by the nodes, while the number of interactions is indicated by the thickness of the lines between them. **F** A bubble diagram of each cuproptosis gene in ICC clusters. **G** A dynamic tree-pruning was used to combine genes with comparable cuproptosis-score patterns into a single module, resulting in a hierarchically clustered tree. **H** Correlation coefficients and matching p-values between CUPT score (CUPTS) and each gene module are shown in boxes

The GSE138709 single cell RNA sequencing dataset was used to analyze the immune microenvironment of ICC samples. After performing gene filtering, normalization, and principal component analysis (PCA), we classified cell populations into 17 clusters (Fig. 2A). These populations were labeled according to previously reported expression patterns (Fig. 2B), identifying malignant cells, B cells, cholangiocytes, dendritic cells, endothelial cells, fibroblasts, hepatocytes, macrophages, natural killer (NK) cells, and T cells (Fig. 2C, Additional file 1: Fig. S1). Remarkably, the greatest variability between individual samples was seen in the representation of malignant cells and cholangiocytes, indicating prominent patient-specific tumor heterogeneity in ICC. Enrichment analysis revealed numerous differentially expressed genes (DEGs), while more stably expressed marker genes confirmed the correct identification of specific cell types (Fig. 2D). Possible interactions between the various cells present in the tumors were examined by exploring the expression of ligand and receptor molecules. This analysis indicated that most interactions were likely to occur between malignant cells and other cell subsets. Receptor-ligand pairs indicating potential interactions were particularly frequent between immune cells and tumor cells (Fig. 2E).

To explore the representation of various cell types in individual samples we created t-SNE plots for each ICC sample. While most samples contained all nine cell types based on gene expression profiles, certain cell populations were missing from a small subset of ICC samples (Fig. 2F, Additional file 1: Fig. S2). Next, we determined the proportion of genes involved in cuproptosis in each cell. This work identified 2930 DEGs.

### Genes of prognostic relevance in ICC patients identified by WGCNA

We retrieved transcriptome expression patterns for 32 ICC samples and 8 normal samples from the TCGA database. For further analysis, we employed the R package edgeR to assess the differential expression of mRNAs between ICC samples and normal tissues. The criteria used for defining differential expression were |log fold change (FC)|> 1 and *P* < 0.05. The genes exhibiting differential expression were utilized to conduct WGCNA analysis. This approach detected several cuproptosis-related prognostic hub genes (Fig. 2G, Additional file 1: Fig. S3A, B). According to the clustering tree and the adjacency and topological overlap matrices, 11 non-gray modules reflecting multiple genes were identified (Fig. 2H). The cuproptosis score highly correlated with the blue module containing 1077 genes, the magenta module, which consisted of 410 genes, and the purple module, which had 721 genes.

### Construction and validation of a cuproptosis-related prognostic signature

**Fig. 3:**
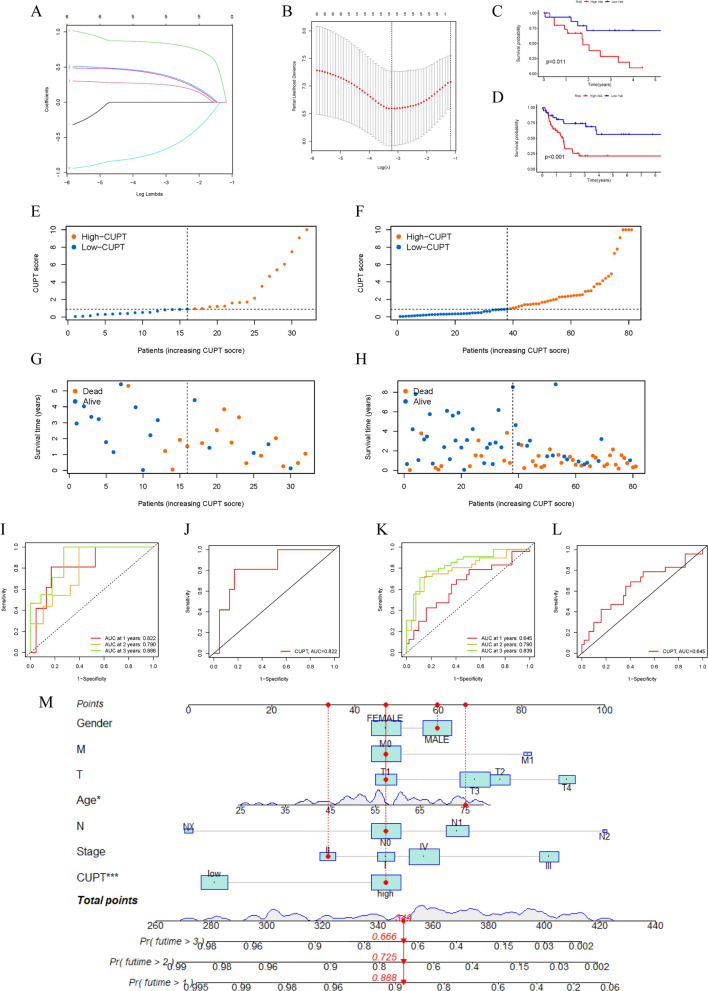
Construction and validation of a cuproptosis-related prognostic signature. **A**, **B** LASSO regression analysis of prognosis-associated genes in ICC. **C**, **D** Comparison of overall survival between high and low-risk groups shown using Kaplan–Meier curves in the TCGA (**C**) and GEO cohort (**D**). **E**, **F** Gene signature-related CUPT score distribution in each patient in the TCGA (**E**) and GEO cohort (**F**). **G**, **H** Survival time of patients ranked in the order of increasing CUPT scores values in the TCGA (**G**) and GEO cohort (**H**). **I**,** J** 1-, 2-, and 3-year survival ROC curves of the prognostic signature based on TCGA (**I**) and GEO cohort data (**J**). **K**, **L** AUC of risk assessment in the TCGA (**K**) and GEO cohort (**L**). **M** The overall survival of patients over the course of 1 to 3 years, predicted using a nomogram, depicting the link between variables in the prediction model

To identify cuproptosis-related genes, we performed an intersection of the hub genes obtained from the single-cell RNA data with the gene expression data from bulk samples collected from the TCGA database. This analysis revealed 307 genes that exhibited a correlated expression pattern (Additional file 1: Fig. S3C). Subsequently, we investigated the relationship between the expression levels of these interlinked genes and overall patient survival. Following an initial screening, we selected six genes to assess their predictive value in the survival of ICC patients (Additional file 1: Fig. S3D). By employing LASSO and Cox analyses for optimization (Fig. 3A, B), we successfully identified three cuproptosis-related genes, namely PLOD2, TNFAIP8, and SLC39A4, which demonstrated a correlation between changes in expression levels and survival data. This analysis allowed us to construct a cuproptosis-related prognostic signature using the following formula: CUPT score = PLOD2*0.6227−TNFAIP8*1.0674 + SLC39A4*0.6969.

Using the median CUPT score to divide patients, individuals were placed into a high or a low CUPT score group. Initial analysis of the training data derived from the TCGA dataset indicated that patients with low CUPT scores showed improved survival (*P* = 0.011). We validated this finding using additional ICC samples and linked data from the GEO database. The analysis of this cohort of samples confirmed the validity of using the CUPT score to predict patient survival (*P* < 0.001) (Fig. 3C, D). When patients were ranked according to their CUPT score, and survival figures were plotted against this ranking, higher scores were clearly associated with shorter survival on the scatter plots using both the training dataset (Fig. 3E) or the validation dataset (Fig. 3F). The distributions of ICC were displayed by ICC curves for the training cohort (Fig. 3G) and validation cohort (Fig. 3H), with an increase in the number of ICC patients. The model had strong prognostic performance, supported by the 1-, 2-, and 3-year AUC values (0.822, 0.790, and 0.886) (Fig. 3I). The analysis of ROC in the validation cohort also supported this conclusion, with 1-, 2-, and 3-year survival rate ROC values at 0.645, 0.790, and 0.839, respectively (Fig. 3J). Additionally, in the training cohort and the validation cohort, the AUC was 0.822 and 0.645 (Fig. 3K, L). Thus, we created a nomogram combining clinicopathological prognostic variables and the CUPT score (Fig. 3M). These findings indicated that the cuproptosis-related prognostic gene signature had significant potential in predicting clinical outcomes in ICC.

### Correlations between CUPT score, somatic mutations, and TMB

**Fig. 4:**
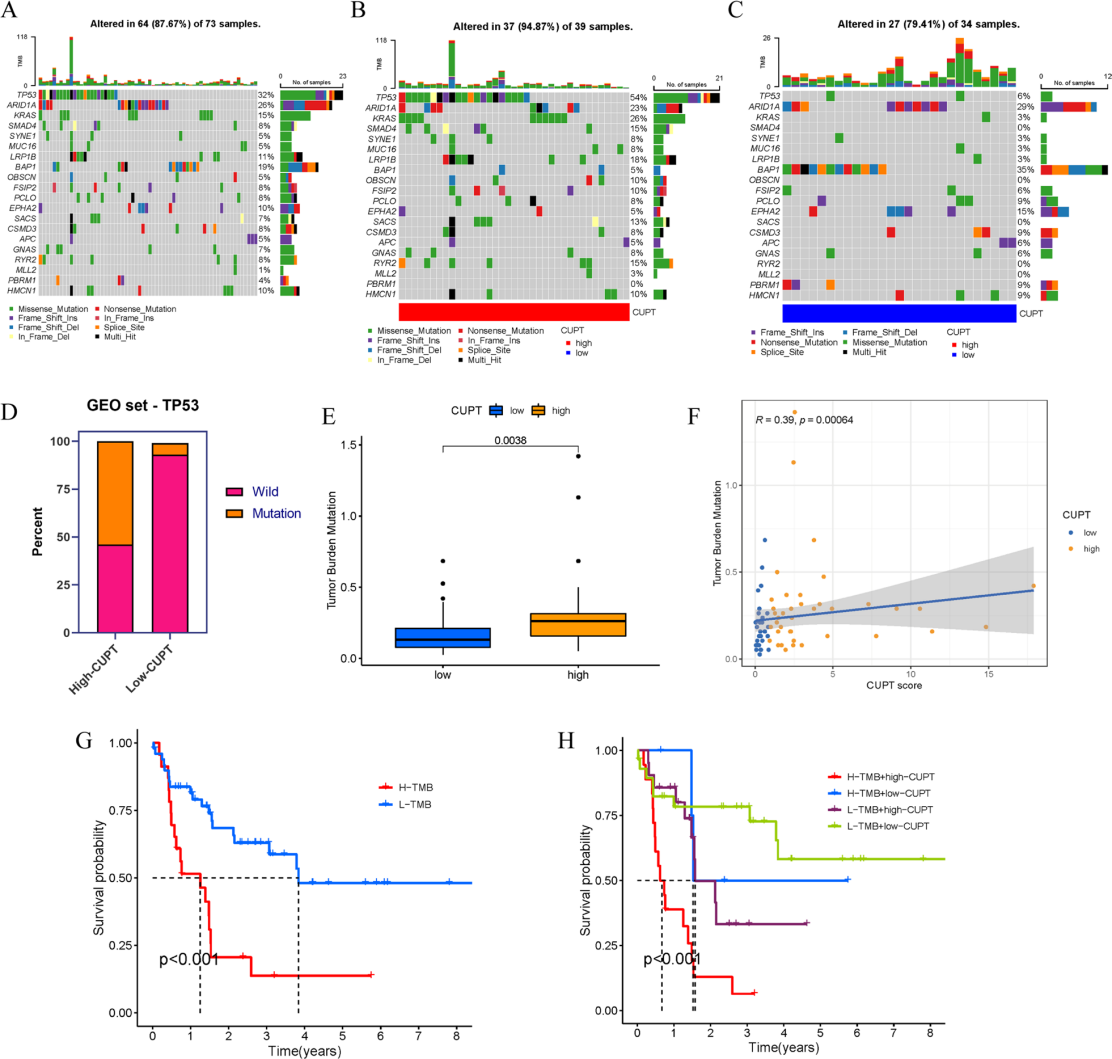
Association between CUPT score, tumor mutation burden (TMB) and somatic mutations. **A**–**C** Waterfall map showing the top 20 genes with the highest rates of mutations. **D** TP53 mutation frequencies in the high and low CUPT score group shown in a bar plot. **E** Differences in TMB between the high and low CUPT score patients (*P* = 0.0038). **F** Scatterplots displaying positive and negative correlations between CUPT score and TMB. **G** Kaplan–Meier curves demonstrating the difference in overall survival between TMB subgroups. **H** TMB and CUPT score stratified Kaplan–Meier curves demonstrating differences in overall survival

Next, we analyzed the frequency of somatic mutations in ICC samples. According to a waterfall map, 64 out of 73 ICC samples (87.67%) contained somatic mutation(s) (Fig. 4A). We then compared the distribution of somatic mutation in ICC patients with high- and low-CUPT scores. To present these data, we selected the top 20 genes most frequently affected by mutations. This analysis revealed that the average frequency of mutations was markedly higher in patients with high CUPT scores (Fig. 4B, C). Of particular note, we observed a significant and striking contrast in the occurrence of TP53 mutations. While 54% of high CUPT score patients carried TP53 mutations, this figure in the low CUPT score group was only 6% (Fig. 4D).

We also evaluated the difference in TMB between high and low CUPT score ICC samples. These comparisons demonstrated that the TMB in the high CUPT group was considerably higher (*P* = 0.0038) (Fig. 4E). Additional correlation analysis also supported a link between CUPT score and TMB (*P* = 0.0006) (Fig. 4F). We analyzed the prognostic value of TMB and found marked differences, with a high TMB correlating with poorer chances of survival (Fig. 4G). Although the result suggested that most patients with a high CUPT score also had high TMB, we conducted a stratified prognostic analysis, exploring the possibility that the simultaneous presence of a high TMB and a high CUPT score acted synergistically in predicting prognosis. In this analysis, patients with a combined low CUPT score and low TMB had the best chances of survival (Fig. 4H). The findings suggested a synergism, indicating that the combination of CUPT score and TMB could improve prognostic predictions.

### Overview of immune infiltration and the distribution of immune cells in ICC patients

**Fig. 5:**
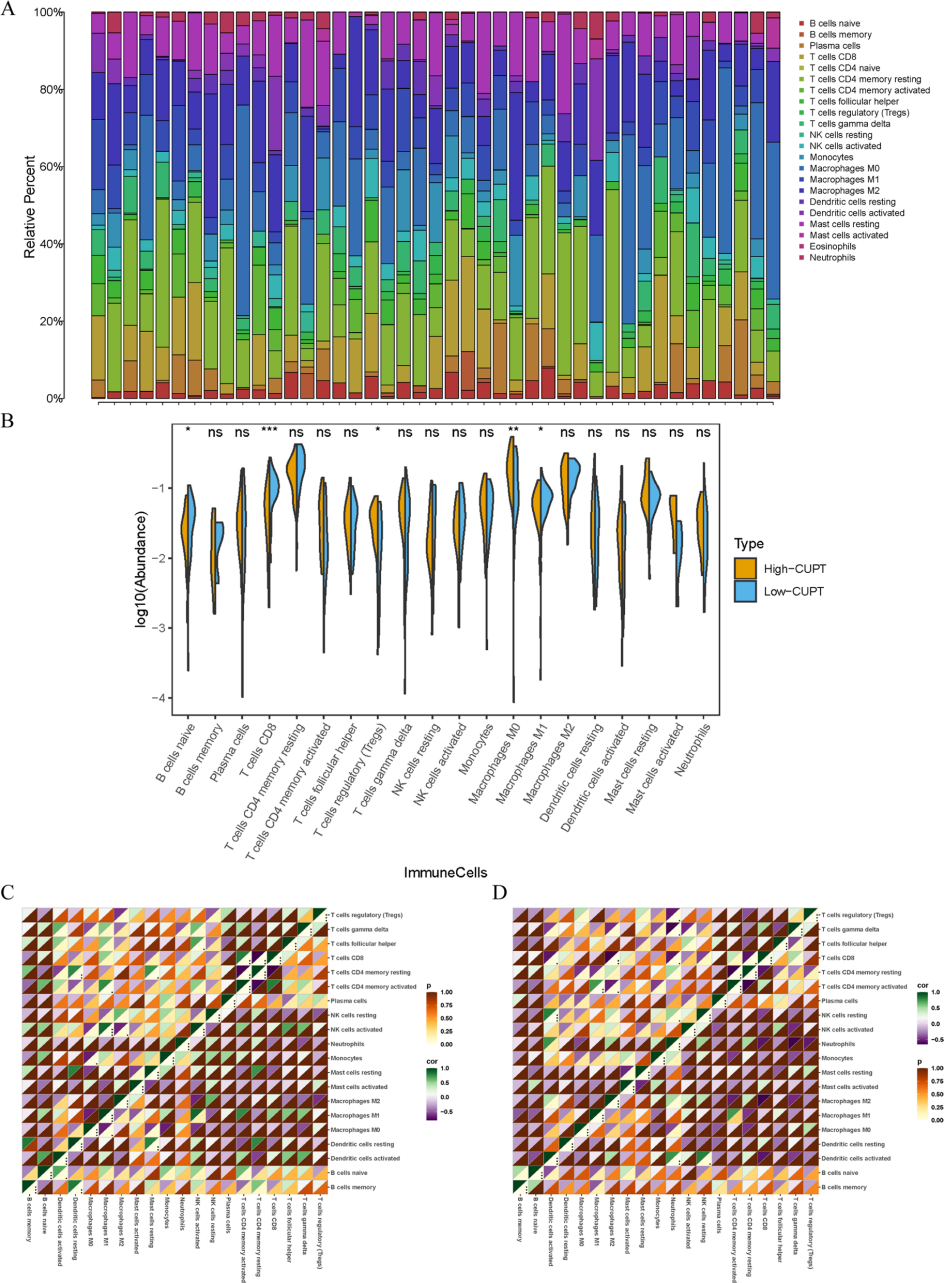
Overview of immune cell infiltration and distribution in ICC patients. **A** Immune cell distribution in patients with high and low CUPT scores. **B** Boxplot illustrating the abundance of the immune cells in high and low CUPT score groups. **C**, **D** Correlation analysis of the immune cells. The correlation is represented by the upper triangle, and the coefficient is shown by the lower triangle

Since the single-cell sequence data contained a lot of information on immune cells, we analyzed the infiltrating immune cells in ICC the CIBERSORT algorithm was used to categorize these cells (Fig. 5A). In this figure different colors represented distinct immune cell populations while the length of the bars is proportional to the frequency of the cells within that category. This analysis revealed that tumors in the high CUPT score group contained significantly lower numbers of naïve B cells, CD8 T cells, and M1 macrophages. At the same time, these tumors had higher levels of regulatory T cells (Tregs) and M0 macrophages (Fig. 5B). Representing the data as a histogram of immune cells showed that the high CUPT score group was characterized by low CD4 memory T cell numbers and low resting CD8 positive T cell abundance, whereas the number of activated NK cells and M1 macrophages was high (*P* < 0.05, Fig. 5C, Additional file 2: Table S2). In contrast, in the low CUPT group, there was a strong positive relationship with resting NK cells and activated dendritic cells, while CD8 positive T cells and M2 macrophage numbers showed a negative correlation (*P* < 0.05, Fig. 5D, Additional file 2: Table S2).

### Gene set enrichment and potential therapeutic value

**Fig. 6:**
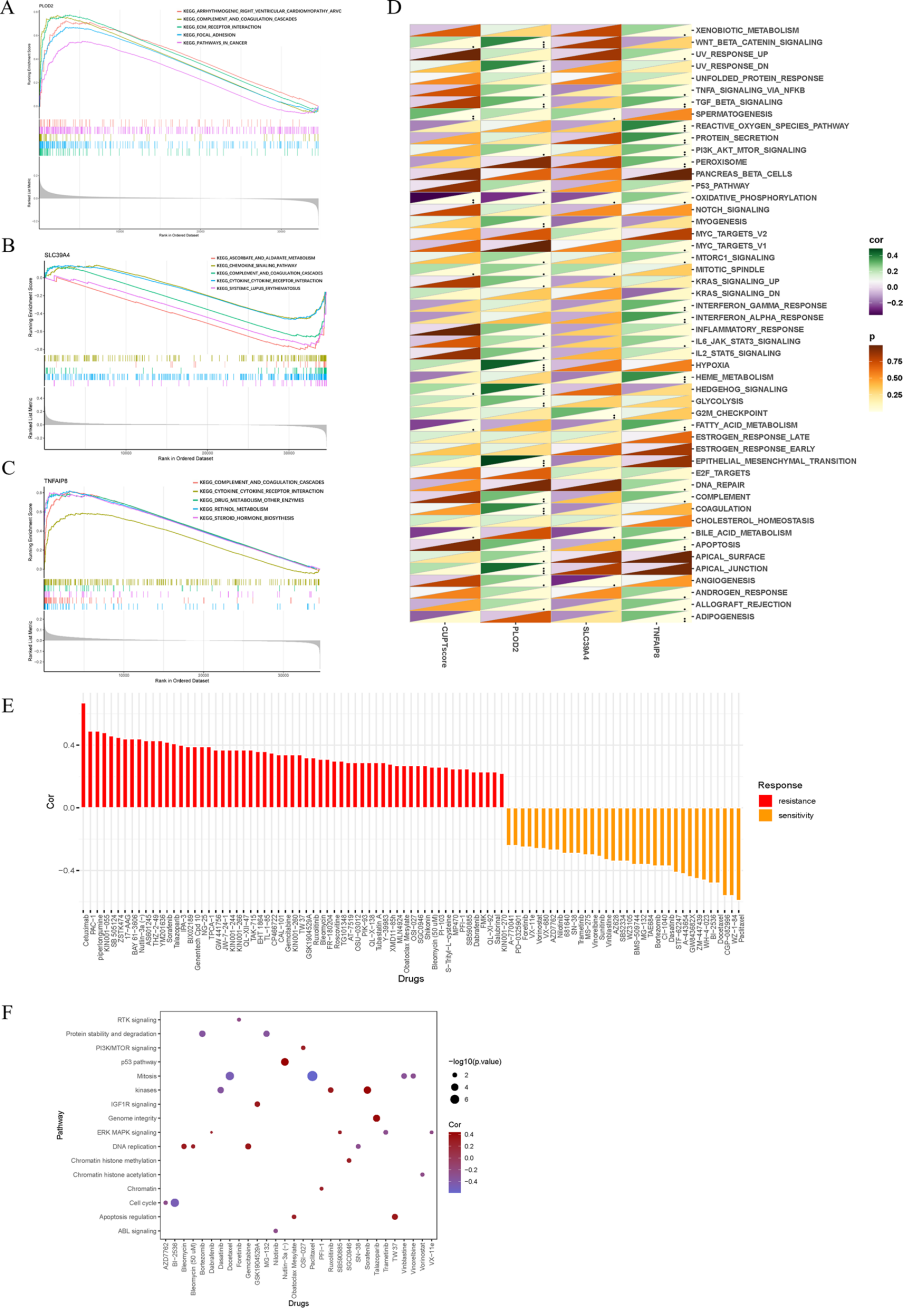
Gene set enrichment and potential predictive therapeutic value. **A**–**C** KEGG functional pathways enriched in the gene matrices of PLOD2, SLC39A4, and TNFAIP8 in the high and low CUPT score groups shown in multi-GSEA plots. Functional pathways enriched in gene matrices of the ICC patients in the high CUPT score group are represented by the curves above the X-axis while pathways enriched in low CUPT score ICC patients are represented by curves below the X-axis. **D** Correlation analysis of HALLMARK pathways. The upper triangle represents the correlation, and the lower triangle represents the coefficient. **E** Bar plot illustrating the correlation between CUPT score and drug sensitivity. **F** Dot plot showing the pathways targeted by medications correlated with the CUPT score

To explore the key biological functions of the three cuproptosis-related genes that contributed to the calculation of the CUPT score (PLOD2, TNFAIP8, SLC39A4) we conducted GSEA and GSVA analyses. PLOD2 appeared to be highly enriched in arrhythmogenic right ventricular cardiomyopathy and in the complement and coagulation cascades according to GSEA results and functional KEGG pathway analysis (Fig. 6A). SLC39A4 was mainly enriched in ascorbate and aldarate metabolism and TNFAIP8 was enriched in complement and coagulation cascades (Fig. 6B, C). In GSVA analysis, PLOD2 and TNFAIP8 were abundant in HALLMARK pathways, while SLC39A4 was enriched in spermatogenesis, oxidative phosphorylation, mitotic spindle formation, and bile acid metabolism (Fig. 6D).

To establish a relationship between CUPT score and the half-maximal inhibitory concentration (IC50) of anticancer medication tested in cell lines, we carried out Spearman correlation analyses. A total of 95 different drugs were identified that were linked CUPT score (Fig. 6E); 34 of these showed drug sensitivity linked to the risk score, and 61 drugs were offered that have drug resistance tied to a risk score. Additional file 2: Table S3 provides a summary of additional medications linked to the CUPT score. The correlations of paclitaxel and cetuximab with CUPT score were 0.59 (*P* < 0.0001) and 0.67 (*P* < 0.0001), respectively, and drug sensitivity differed significantly between CUPT high and low groups (*P* < 0.0001) (Additional file 1: Fig. S4A–D). Gemcitabine, a commonly used medication in the clinical routine of ICC, demonstrated a significant correlation with CUPT (*P* = 0.0018). Additionally, the findings revealed that tumors in high CUPT score patients exhibited heightened sensitivity to this drug (Additional file 1: Fig. S4E, F). The molecular pathways targeted by these medications are shown in Fig. 6F and Additional file 2: Table S4. We discover that medications with high sensitivity and high CUPT scores commonly affected the p53 pathway, kinases, and genome integrity, while treatments with low CUPT scores are more likely to target mitosis, the cell cycle, and kinases. From these findings it appears that the CUPT score may be a usable biomarker in predicting medication responses in ICC.

### Modeling gene selection and survival analysis

**Fig. 7:**
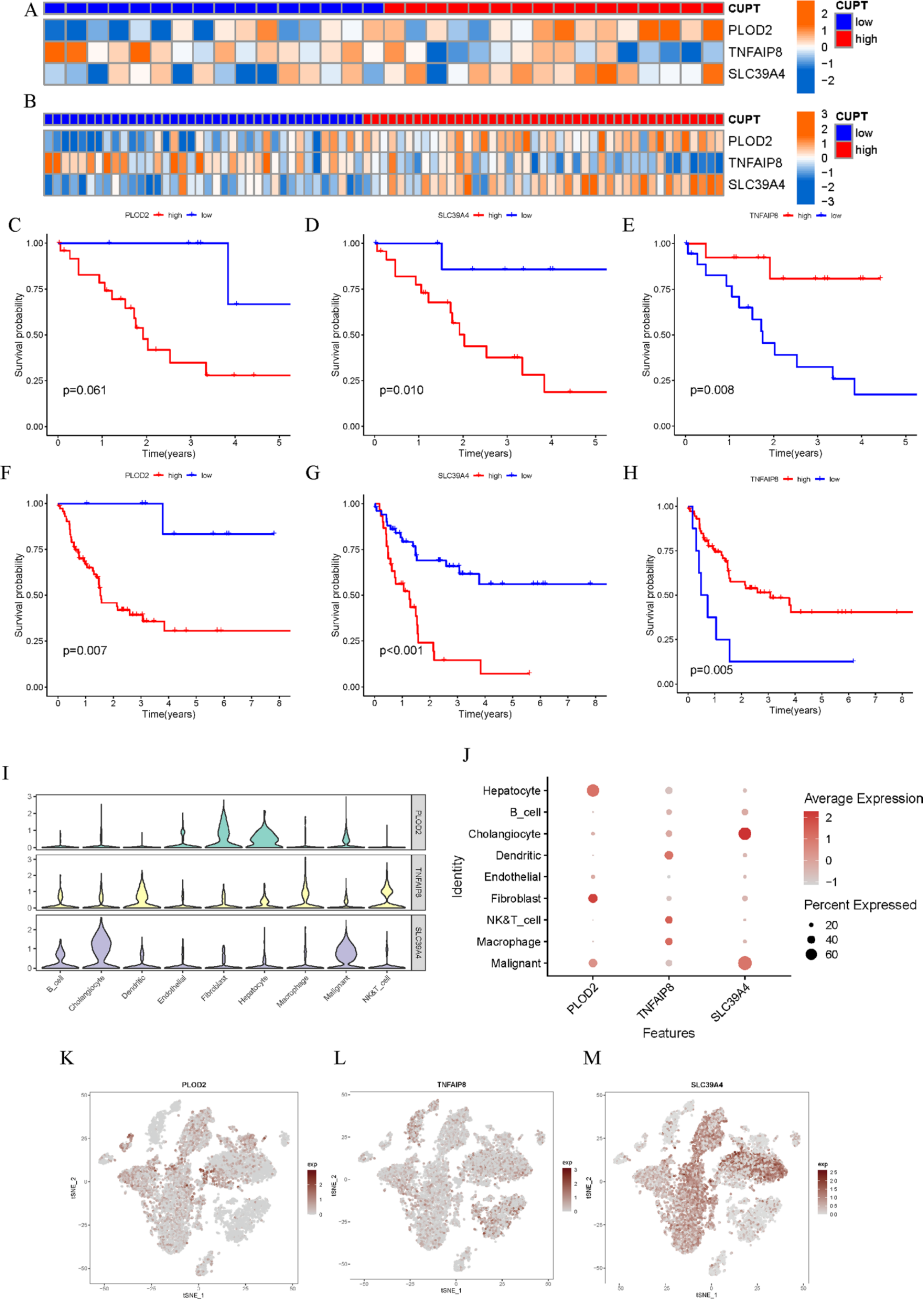
Modeling of gene selection. **A**, **B** Heatmaps displaying the expression of three cuproptosis-related prognostic genes from the TCGA (**A**) and GEO dataset (**B**). **C**–**H** Kaplan–Meier curves showing overall survival differences based on PLOD2, SLC39A4, and TNFAIP8 expression in the high- and low- risk groups in the TCGA (**C**–**E**) and GEO data (**F**–**H**). **I**,** J** Three modeling genes for each cell type are displayed in ICC using a violin plot and bubble chart. **K**–**M** The proportion of each cell’s three modeling genes

We assessed the expression of three cuproptosis-related prognostic genes in the training and validation cohorts in order to choose the modeling gene. The expression patterns of three cuproptosis-related prognostic genes were displayed in the heatmaps for the training cohort (Fig. 7A) and validation cohort (Fig. 7B). According to these findings, PLOD2 and SLC39A4 were expressed more in high CUPT score ICC lesions, whereas TNFAIP8 was expressed less than in the low CUPT score group. We then identified three genes that were related to ICC patient prognosis. In both the training and validation cohorts, the prognosis of patients improved considerably with low PLOD2 and SLC39A4 expression, while TNFAIP8 demonstrated the opposite tendency (Fig. 7C–H). The expression of modeling genes in various cell types was then investigated using single-cell sequencing samples. As shown in Fig. 7I–M, PLOD2 was mainly expressed in hepatocytes and fibroblasts, TNFAIP8 was mainly expressed in dendritic cells, NK cells, T cells, and macrophages, while SLC39A4 was primarily expressed in cholangiocytes and malignant cells.

The HR value of SLC39A4 was the highest in Cox regression and LASSO regression analyses (HR = 2.008, *P* = 0.02). Survival data also showed that patients with high SLC39A4 expression had a considerably worse prognosis (*P* < 0.001, Fig. 4D, G). Moreover, SLC39A4 expression in cholangiocytes and malignant cells was considerably higher than the abundance of PLOD2 and TNFAIP8 transcripts. Based on these results, we decided to analyze the role of SLC39A4 experimentally.

### Verification of the cuproptosis-related prognosis signature in ICC cells

**Fig. 8:**
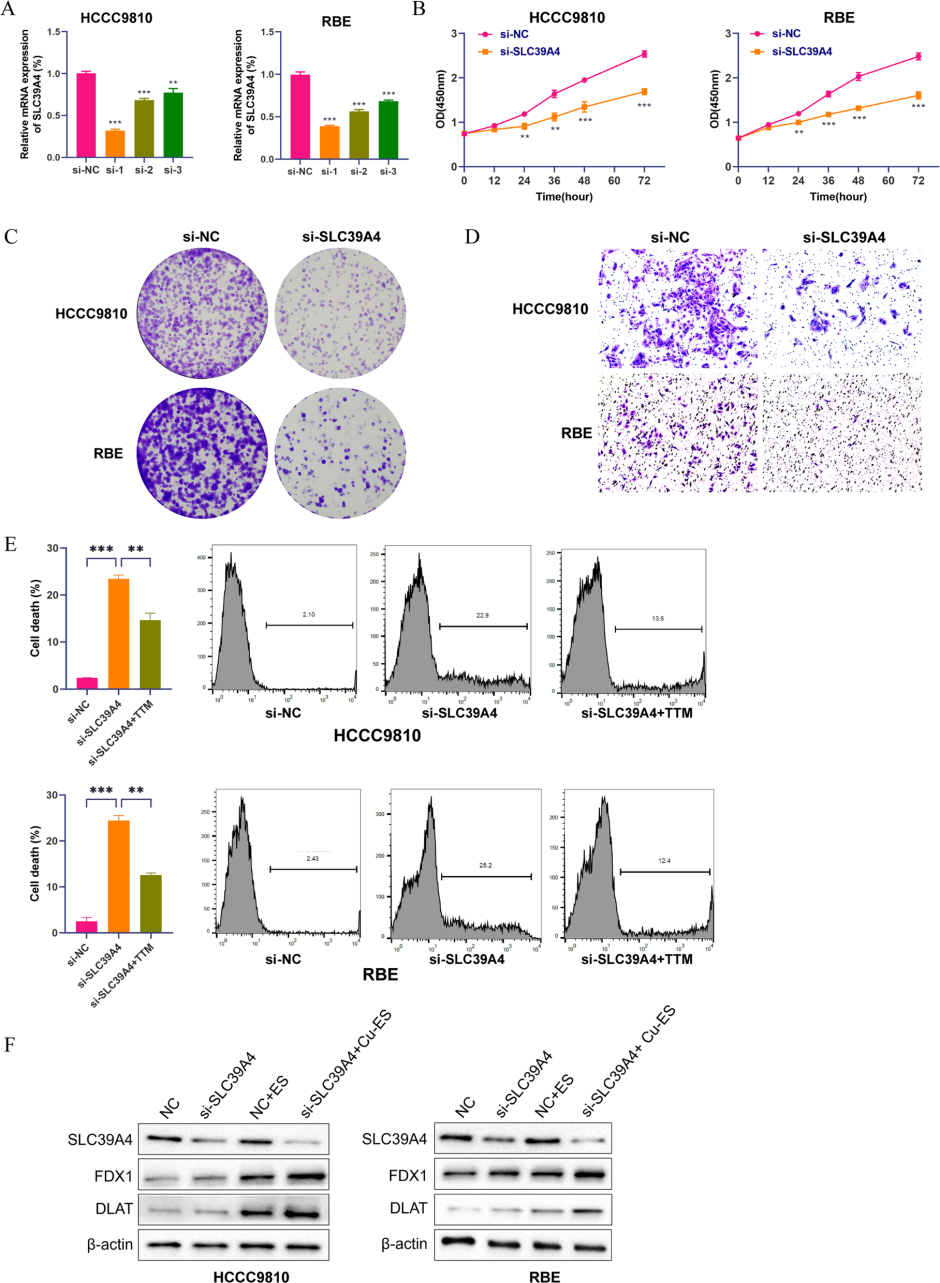
Verification of the cuproptosis-related prognosis signature in ICC cells. **A** mRNA levels of SLC39A4 48 h after transfection. **B** Proliferation of ICC cells detected by CCK8 assay. **C** Colony formation assays of ICC cells after siRNA transfection. **D** Results of migration assays of ICC cells in Transwell plates after siRNA transfection. **E** Results of ICC cell death after adding TTM.** F** Protein expression of key genes of cuproptosis after knockdown of SLC39A4 and addition of ES

To explore the role of SLC39A4 expression in influencing the biological behavior of ICC cells, we conducted siRNA knockdown experiments in HCCC9810 and RBE cell lines in vitro. SLC39A4-specific siRNAs were designed and produced, and both cell lines were transfected with these reagents. Following a 48 h incubation period after the transfection, qRT-PCT assays showed that the siRNA-1 sequence caused a considerable downregulation in SLC39A4 mRNA expression (*P* < 0.001) (Fig. 8A). When the viability of the transfected cells was tested in CCK8 assays, it was apparent that SLC39A4 knockdown reduced the capacity of the treated HCCC9810 and RBE cells to proliferate (Fig. 8B). We also tested the effect of the gene knockdown on the ability of seeded tumor cells to form new colonies. Compared to non-transfected controls, siRNA treated HCCC9810 and RBE cells formed colonies less efficiently (Fig. 8C). Furthermore, in a Transwell migration assay the reduction in SLC39A4 expression also reduced the invasive capacity of both cell lines (Fig. 8D). To investigate the effect of SLC39A4 knockdown on cell death in ICC, we assessed cell death after silencing SLC39A4. The results revealed an increase in cell death following SLC39A4 knockdown. However, the addition of a copper chelator: tetrathiomolybdate (TTM) effectively suppressed ICC cell death and restored cellular viability (Fig. 8E). Western blotting of the transfected cells showed that the knockdown of SLC39A4 increased the protein levels of key genes of cuproptosis: FDX1 and DLAT. After treating with 20 nM of Cu-elesclomol (Cu-ES) for 24 h, there was a significant increase in the expression of FDX1 and DLAT. Furthermore, knockdown of SLC39A4 resulted in a further increase in the expression of FDX1 and DLAT (Fig. 8F). The results presented above highlight a significant correlation between viability, colony forming capacity, migration and cell death induced by siRNA transfection and cuproptosis processes.

## Discussion

As early clinical manifestations of ICC are subtle and non-specific, diagnosing these tumors is extremely challenging [26]. At the time of the eventual detection, in most patients ICC has already spread to such an extent that any opportunity for radical surgical resection is lost. It has been widely reported that ICC is resistant to radiotherapy, mainly due to the unique tumor microenvironment (TME) associated with this malignancy [27, 28]. This unusual TME shows abundant signs of mesenchymal fibrosis and pronounced infiltration by immune cells and tumor-associated fibroblasts. Apart from promoting tumor growth, this highly fibrotic environment also renders tumors more treatment resistant [29]. The variability of TME is a key determinant of tumor behavior. During the interplay between malignant cells and surrounding stromal cells mediators supporting the development of immune tolerance may be released. In other cases, the infiltrating immune cells in the microenvironment can kill tumor cells, preventing their continued proliferation and metastasis [30]. Therefore, exploring the immune microenvironment in ICC has become a focus of interest in our studies. The possibility that the immune microenvironment may be altered during the process of cuproptosis also supported the need for further studies in this area.

The work presented here proposes a prognostic model designed to categorize ICC patients based on the expression levels of specific cuproptosis-related genes. The calculated CUPT score divided patients into high or low CUPT score subsets. Analyzing Kaplan–Meier survival curves from patients in the TCGA and GEO databases identified statistically significant differences in the length of survival between the high and low CUPT score groups. These findings provided preliminary support for the utility of our model in predicting clinical outcomes in ICC. The AUC at 1, 3, 5 years were 0.822, 0.790, and 0.886 in TCGA cohort and 0.645, 0.839, and 0.807 respectively in the GEO cohort of patients, supporting the accuracy of our proposed model. The PCA analysis results indicated that the patients in the high and low CUPT scoring groups were separated into distinct dimensions, thereby confirming the model's ability to accurately differentiate between patients. To predict survival in ICC, we developed a nomogram that incorporated both established pathological risk factors and the CUPT score. This nomogram demonstrated exceptional accuracy in predicting clinical outcomes.

Next, we explored the role of TME and the somatic mutation map in ICC patients. The analysis of somatic mutations revealed a high frequency of mutations affecting the ARID1A and EPHA2 genes, both in high and low CUPT score patients. Guo et al. [31] previously reported that most ARID1A mutation inhibited TGF-β mediated signaling pathways, potentially playing a causative role in the development of CAA. Furthermore, immune checkpoint inhibitor-induced antitumor immunity was reported to be more pronounced in patients with ARID1A loss [32]. Mutations affecting EPHA2 also represent a potential novel therapeutic target during lymphatic metastasis of ICC [33]. We found statistically significant differences in the frequency of TP53 mutations in patients with high or low CUPT scores, although TP53 mutations are very common, occurring in more than 50% of human tumors [34–36]. The integrin-FAK-SRC pathway represents a potential mechanism by which TP53 mutations may be involved in ICC metastasis formation [3]. Boerner et al. [37] also reported that TP53 mutations could predict a poorer prognosis in patients with unresectable ICC. These findings suggest that TP53 mutations may represent a clinically significant feature in high-risk patients. TMB is emerging as a possible biomarker and may have utility in predicting the effectiveness of immune checkpoint inhibitor therapy [38]. Patients with elevated TMB respond well to immunotherapy, often irrespective of the origin of their tumors. In our study, TMB was noticeably higher in patients with high CUPT scores. Moreover, the 5-year survival was the lowest in the group of patients where a high-CUPT score coincided with a high TMB.

The composition of the immune cell infiltrate was also different between the high and low CUPT score groups. Yang et al. [39] previously reported an enrichment of M0 macrophages in ICC tissues, potentially influencing the clinical prognosis of the disease. In another study, a subset of ICC patient was found to have a significantly increased number of CD8^+^ T cells in their tumors, and this phenomenon was associated with a favorable outcome [40]. Additionally, M1 macrophages have the capacity to modify the immune milieu by recruiting additional macrophages and NK cells to the TME and inducing their activation locally. Thus, it was proposed that this behavior of M1 macrophages may be exploited in the immunotherapy of the disease [41]. In our experiments patients in the high CUPT group had significantly more M0 macrophages in their tumors, while in low CUPT patients CD8 positive T cell numbers were notably increased. Interestingly, there was also a positive correlation between NK cells and M1 macrophages in the high CUPT group. This suggests that M1 macrophages may have the potential to alter the immune microenvironment in ICC by recruiting and subsequently activating NK cells. Again, if confirmed in further studies, this mechanism could be exploited in the immunotherapy of ICC.

In advanced ICC a combination of surgery and chemotherapy represents the main treatment option, although drug resistance is a major factor in treatment failure, resulting in eventual mortality. Studies have shown that the simultaneous administration of copper complexes and chemotherapeutic drugs acts synergistically, enhancing drug responses. Copper complexes appear to play a particularly important role during treatment with platinum containing therapeutic agents by influencing regulatory processes involved in copper homeostasis [11]. Systemic chemotherapy is the most commonly utilized treatment modality in patients with unresectable ICC [42]. In this context we found that high CUPT score patients may be more sensitive to cetuximab, while patients with low CUPT scores potentially respond better to treatment with paclitaxel. Furthermore, we also investigated the relationship between the drug sensitivity of gemcitabine and the CUPT score. Gemcitabine, a classical chemotherapeutic drug used in the treatment of CAA, can be effective in some cases of ICC [43]. However, its therapeutic effectiveness depends on the pathologic tumor type, the stage of the lesion, and the physical status of the patient. Gemcitabine is a deoxypyrimidine analogue that exerts its antitumor effects by inhibiting cellular DNA synthesis. After entering the cells, the drug is phosphorylated to produce its active nucleoside diphosphate form that inhibits the extension of DNA strands. This mechanism causes DNA breakages and apoptosis [44]. In our study, we demonstrated a correlation between CUPT scores and gemcitabine sensitivity, with patients in the high CUPT score group responding well to gemcitabine treatment. In addition, drugs targeting the mitotic process, cell cycle progression, and certain kinases may be more effective in patients in the low CUPT score group. In contrast, drugs targeting the p53 pathway or affecting genome integrity may be more effective in patients with high CUPT scores.

Solute carrier family 39 member 4 (SLC39A4) plays an important role in cellular zinc homeostasis [45]. This transporter was shown to accelerate the growth of esophageal squamous cell carcinomas [46]. Interestingly, SLC39A4 expression is constitutionally downregulated in nude mice, reducing the growth and migration of gallbladder cancer cells and delaying the development of transplanted tumors [47]. Despite this observation, the effect of SLC39A4 expression has not been previously reported in human ICCs. In our study, SLC39A4 exhibited the highest HR value in Cox regression and LASSO regression analysis, and survival analysis revealed that patients with high SLC39A4 expression had considerably worse prognosis, suggesting that SLC39A4 might be a biomarker of ICC progression, and could represent a potential therapeutic target. In general biology, SLC39A4 appears to be mainly associated with ascorbate and aldarate metabolism and plays a role in pathways in oxidative phosphorylation, mitotic spindle formation, and bile acid metabolism. Our in vitro experiments confirmed that the knockdown of SLC39A4 using a siRNA reduced the ability of ICC cell lines to proliferate, form colonies, or migrate. Knockdown of SLC39A4 also increased the expression of key genes of cuproptosis: FDX1 and DLAT. When Cu^2+^ accumulates excessively in cells dependent on mitochondrial respiration, Cu^2+^ binds to thioctylated DLAT, inducing heterodimerisation of DLAT. The increase in insoluble DLAT leads to cytotoxicity and induces cell death. FDX1, on the other hand, is involved in regulating the lipoic acidification of DLAT and reduces Cu^2+^ to the more toxic Cu^+^, leading to the inhibition of Fe-S cluster protein synthesis and the induction of cell death [10]. These changes coincided with increased cuproptosis activity and may inhibit metastasis formation by ICC cells. The research conducted by the Tsvetkov team has confirmed that Cu-ES can greatly suppress cell growth and induce cell death, classifying it as a typical cuproptosis stimulator [10]. Interestingly, when TTM is combined with Cu-ES treatment, it does not affect cell growth [48]. Our findings demonstrate that TTM effectively inhibits cell death induced by SLC39A4 knockdown, highlighting the intricate relationship between SLC39A4 and cuproptosis. Moreover, the expression of key proteins involved in cuproptosis, such as FDX1 and DLAT, is further upregulated in ICC cells with SLC39A4 knockdown, providing additional evidence that SLC39A4 knockdown can influence various cellular processes, including proliferation, apoptosis, and migration, through the cuproptosis pathway.

However, the effect of SLC39A4 on ICC cuproptosis remains to be clarified further. Moreover, the specific mechanisms linking cuproptosis-related genes to changes in the TME and to chemotherapy resistance are still unclear. Nevertheless, this study provides the first evidence linking cuproptosis to the biological behavior of ICCs.

## Conclusions

In conclusion, through the analysis of single-cell and bulk RNA sequencing data, we devised and validated a novel prognostic model based on the expression of molecules associated with cuproptosis. In addition, we were able to experimentally confirm the role SLC39A4 in the development of ICC. We also found that the developed CUPT score was able to predict certain features of the infiltrating immune cells, the somatic mutational landscape, and the probable biological behavior of ICC. Some of the described observations may also influence future therapeutic approaches in this disease.

### Supplementary Information

**Additional file 1: Supplementary Figures.**

**Additional file 2: Supplementary Tables.**

## Data Availability

Databases analyzed for this study are available in online repositories. Detailed information can be found in the article.
