# Supplementary material for: A cuproptosis-related gene expression signature predicting clinical prognosis and immune responses in intrahepatic cholangiocarcinoma detected by single-cell RNA sequence analysis
Source: Cancer Cell Int. 2024 Mar 2;24:92. doi: 10.1186/s12935-024-03251-2 (PMC10908169; doi:10.1186/s12935-024-03251-2)
Supplement: Supplementary file 1 — Additional file 1: Supplementary Figures. [file 12935_2024_3251_MOESM1_ESM.docx]

**Additional Figure**


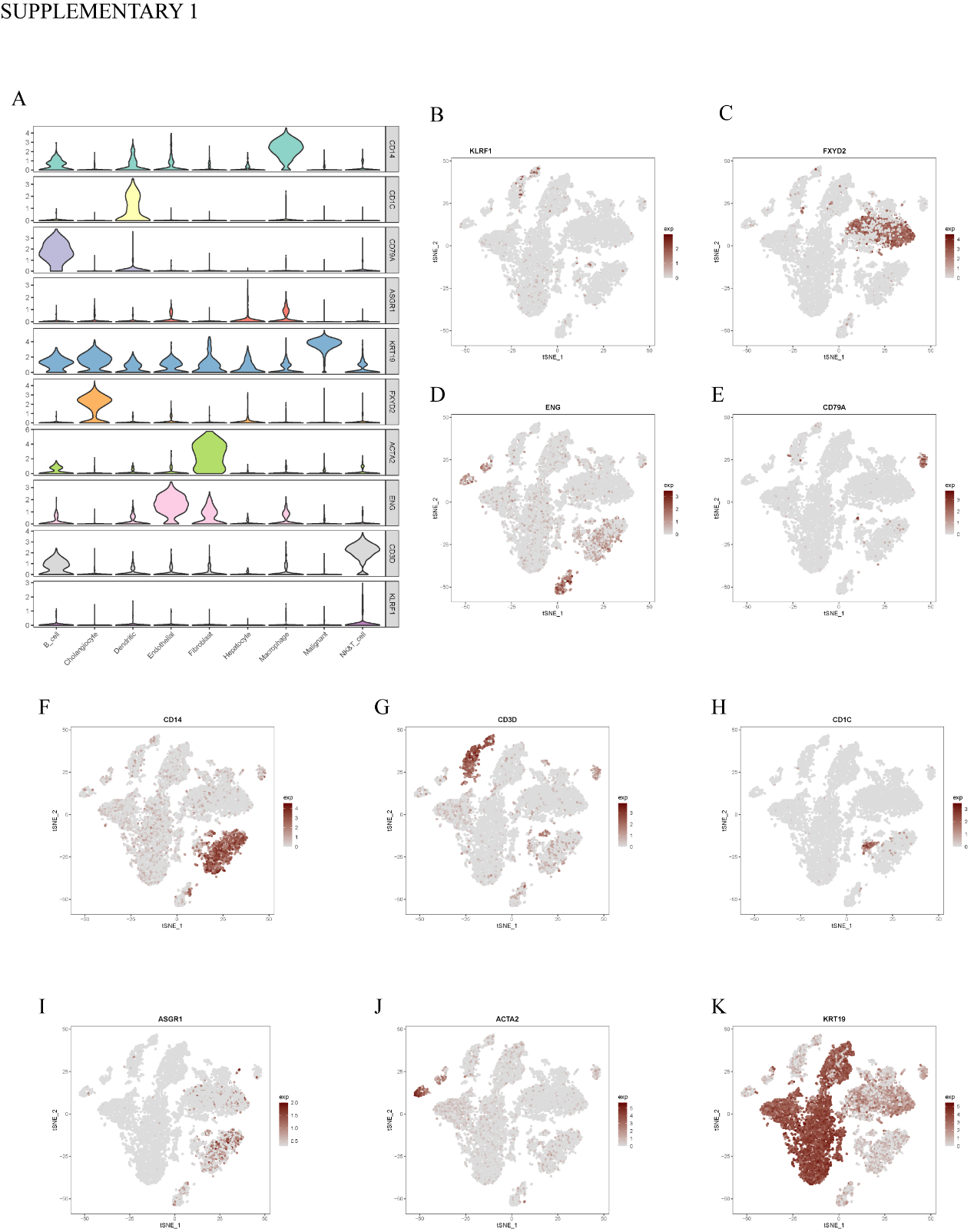


**Additional Figure 1** The expression of canonical marker genes for distinct cell types shown in t-SNE plots. **(A)** Violin plot showing the canonical marker genes of each cluster in ICC. **(B-K)** The expression of canonical marker genes for distinct cell types shown in t-SNE plots.


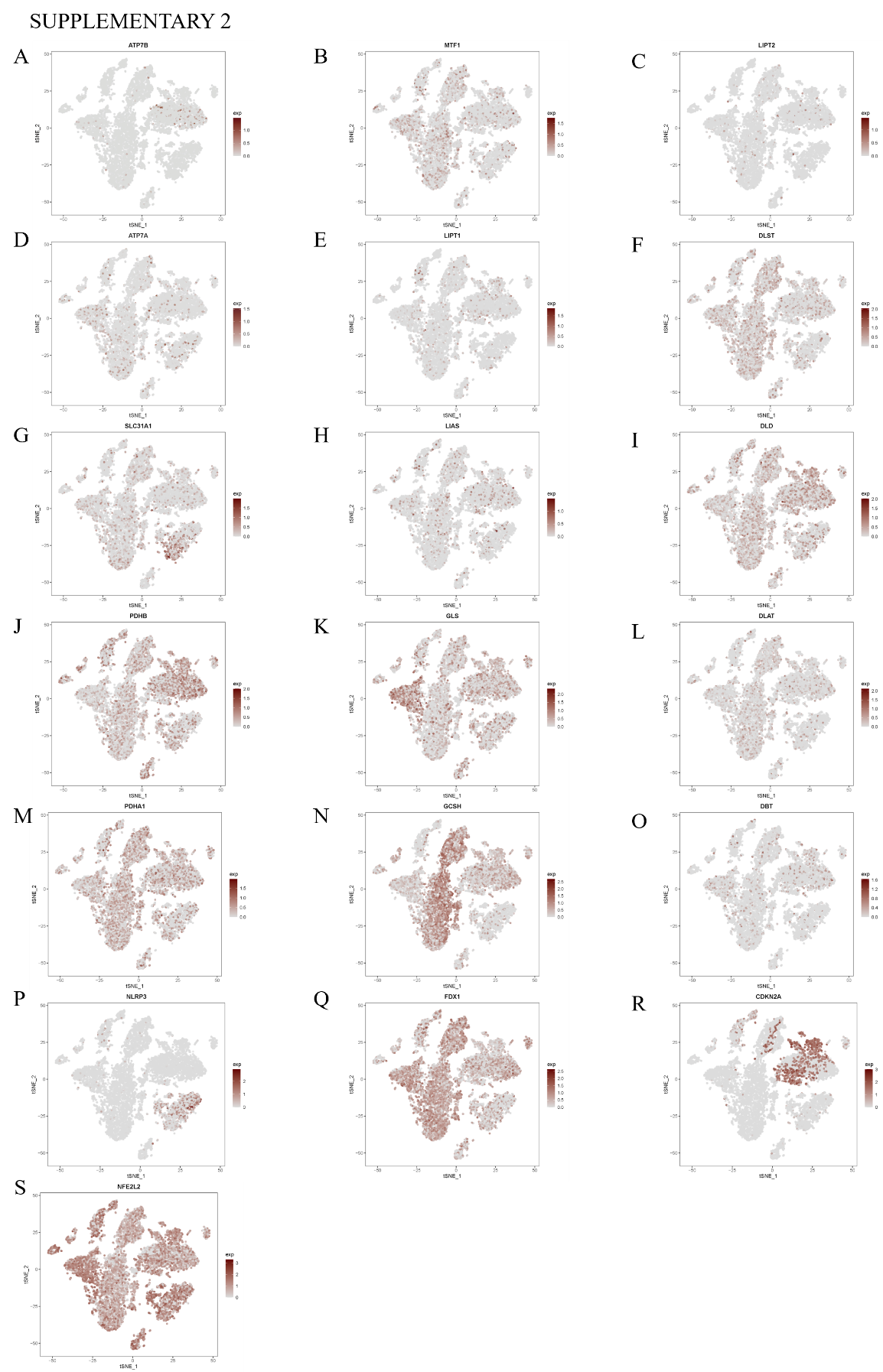


**Additional Figure 2** The expression of cuproptosis-related genes for distinct cell types shown in t-SNE plots. **(A-S)** The expression of cuproptosis-related genes for distinct cell types shown in t-SNE plots.


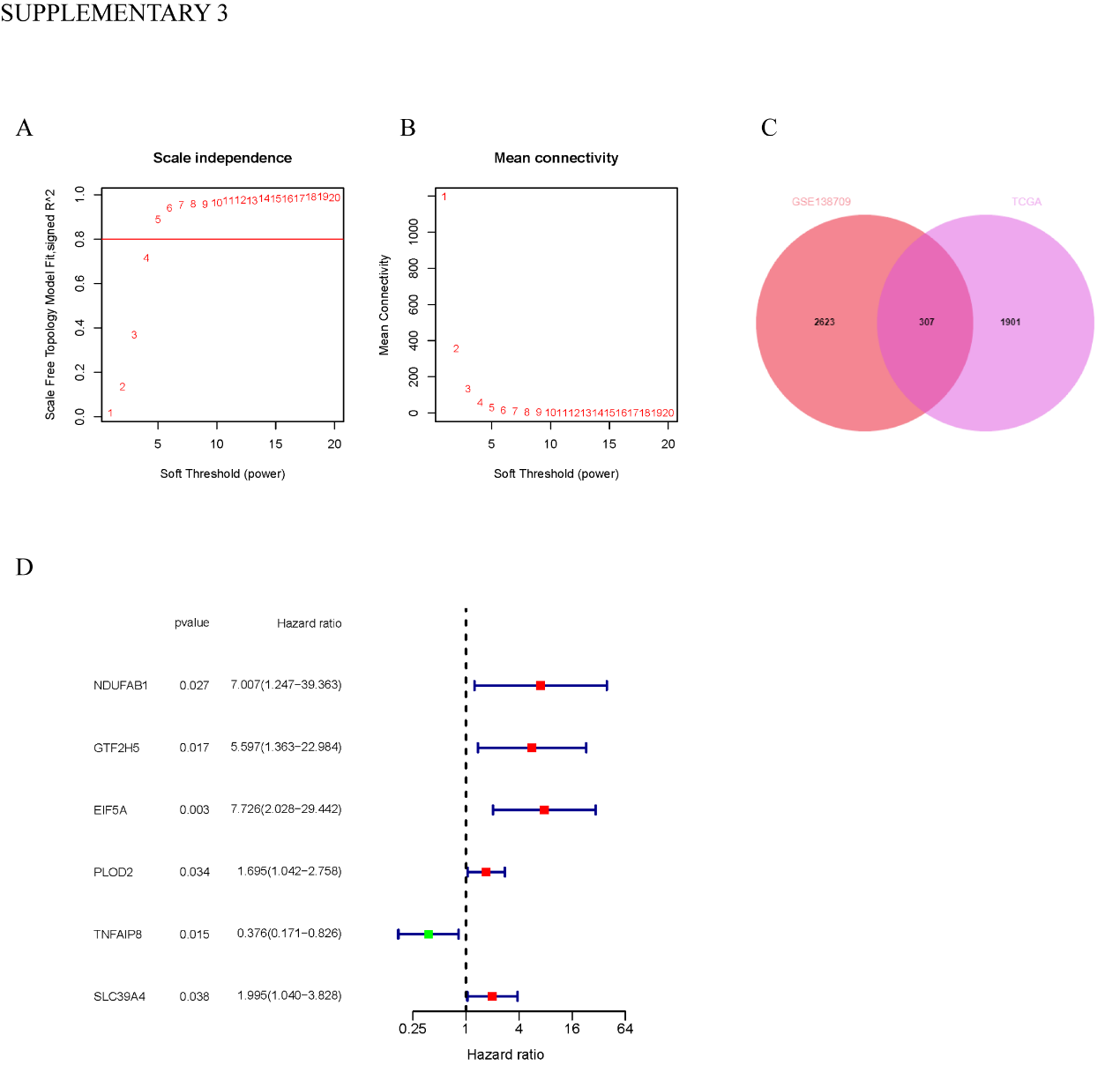


**Additional Figure 3** Construction of cuproptosis-related prognostic signature **(A, B)** Screening of the soft threshold. **(C)** Venn diagram showing 307 co-existing genes for TCGA and GEO dataset. **(D)** Correlation between six-gene expression levels and the risk of survival time.


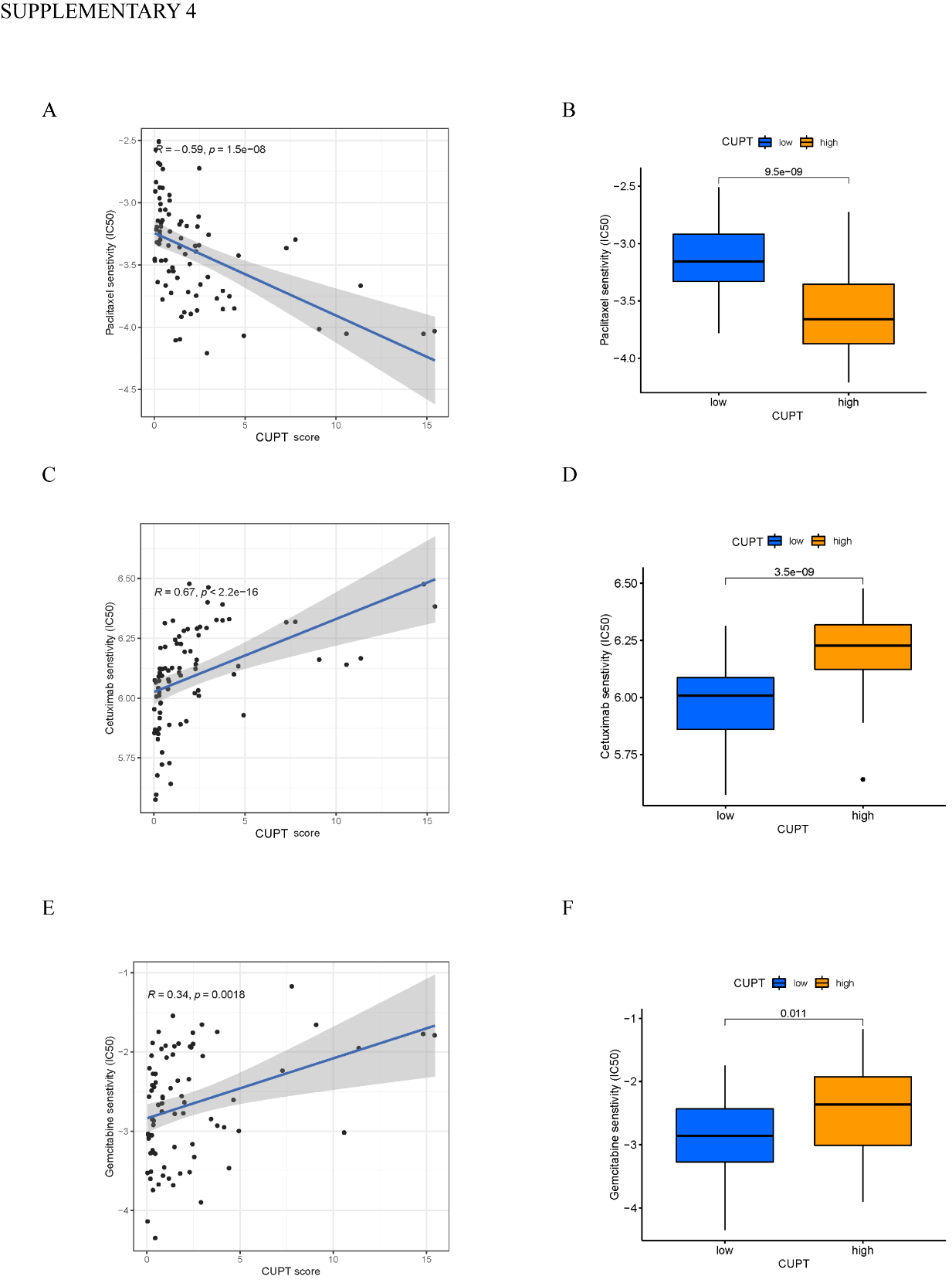


**Additional Figure 4** Relationship between drug sensitivity and CUPT score **(A)** Scatterplots displaying positive and negative correlations between the CUPT scores and responses to paclitaxel (IC50). **(B)** Paclitaxel sensitivity (IC50) variations between high- and low-CUPT groups. **(C)** Scatterplots displaying positive and negative correlations between the CUPT scores and responses to cetuximab (IC50). **(D)** Cetuximab sensitivity (IC50) variations between high- and low-CUPT groups. **(E)** Scatterplots displaying positive and negative correlations between the CUPT scores and responses to gemcitabine (IC50). **(F)** Gemcitabine sensitivity (IC50) variations between high- and low-CUPT groups.
